# Line Transect Surveys Underdetect Terrestrial Mammals: Implications for the Sustainability of Subsistence Hunting

**DOI:** 10.1371/journal.pone.0152659

**Published:** 2016-04-13

**Authors:** José M. V. Fragoso, Taal Levi, Luiz F. B. Oliveira, Jeffrey B. Luzar, Han Overman, Jane M. Read, Kirsten M. Silvius

**Affiliations:** 1 Stanford University, Stanford, CA, 94305–5020, United States of America; 2 Department of Fisheries and Wildlife, Oregon State University, Corvallis, OR, United States of America; 3 Departamento de Vertebrados, Museu Nacional, UFRJ, RJ, 20.940–040, Brazil; 4 Stanford University, Stanford, CA, 94305–5020, United States of America; 5 Environmental and Forest Biology, State University of New York-College of Environmental Science and Forestry, Syracuse, NY, 13210, United States of America; 6 Geography Department, Syracuse University, Syracuse, NY, 13244, United States of America; 7 Department of Forest Resources and Environmental Conservation, Virginia Tech, Blacksburg, VA, United States of America; University of Sydney, AUSTRALIA

## Abstract

Conservation of Neotropical game species must take into account the livelihood and food security needs of local human populations. Hunting management decisions should therefore rely on abundance and distribution data that are as representative as possible of true population sizes and dynamics. We simultaneously applied a commonly used encounter-based method and an infrequently used sign-based method to estimate hunted vertebrate abundance in a 48,000-km^2^ indigenous landscape in southern Guyana. Diurnal direct encounter data collected during three years along 216, four-kilometer -long transects consistently under-detected many diurnal and nocturnal mammal species readily detected through sign. Of 32 species analyzed, 31 were detected by both methods; however, encounters did not detect one and under-detected another 12 of the most heavily hunted species relative to sign, while sign under-detected 12 never or rarely collected species relative to encounters. The six most important game animals in the region, all ungulates, were not encountered at 11–40% of village and control sites or on 29–72% of transects where they were detected by sign. Using the sign methodology, we find that tapirs, one of the terrestrial vertebrates considered most sensitive to overexploitation, are present at many sites where they were never visually detected during distance sampling. We find that this is true for many other species as well. These high rates of under-detection suggest that behavioral changes in hunted populations may affect apparent occurrence and abundance of these populations. Accumulation curves (detection of species on transects) were much steeper for sign for 12 of 16 hunted species than for encounters, but that pattern was reversed for 12 of 16 species unhunted in our area. We conclude that collection of sign data is an efficient and effective method of monitoring hunted vertebrate populations that complements encounter and camera-trapping methods in areas impacted by hunting. Sign surveys may be the most viable method for large-scale, management-oriented studies in remote areas, particularly those focused on community-based wildlife management.

## Introduction

Wild mammals, birds and reptiles are important elements of traditional diets, cultural practices and socio-economic systems in the Neotropics [1, 2]. In the 1980s, documentation of local population depletion of vertebrate species and consequent trophic cascades [3] led to concern that structurally intact tropical forests would lose ecological function due to defaunation caused by hunting (the “empty forest hypothesis” [4]). Subsequent work, based primarily on visual encounter data collected along line transects (distance sampling), has implied that all or most Amazonian forests accessible to humans have suffered significant vertebrate population declines due to overhunting (e.g., [5, 6, 7, 8, 9]). The impact of unsustainable hunting on animal populations is unquestioned; it has been clearly demonstrated in regions affected by the commercial bush-meat trade [10]. The documented challenges [11, 12, 13] in measuring vertebrate abundances in Neotropical forests, however, should alert us to potential weaknesses in our understanding of hunting sustainability in the Amazon, especially in human occupied protected areas where hunting is for subsistence or local exchange purposes.

Because animal population density is an essential parameter for estimating productivity and sustainable harvest rates [5], encounter-based surveys using distance sampling [14] methods became the tool of choice for estimating population densities of terrestrial and arboreal game animals in the 1980s and 90s [6, 15], and they continue to be used (e.g., [16, 17, 18, 19, 20]). Mathematical approaches such as the King method [14] and Distance software [21]) readily enabled estimation of population densities from distance sampling. Transects can also be used to record indirect observation (tracks, feces, other sign), but such data, when collected, are rarely analyzed and published [11, 22] because they cannot be used for density estimation. Occupancy modeling, which provides presence-absence rather than abundance information, is increasingly popular as a population monitoring estimation tool, due in part to the difficulty of obtaining sufficient encounter data for density estimation. Occupancy modeling can incorporate data from a diversity of methods into a single model, including sign, encounter and camera trap data. Camera trapping practitioners are developing or refining density and occupancy estimation models [23, 24], improving the method’s versatility and applicability. Despite increasing adoption of these new methods, however, distance sampling and density estimation using transect based data remain preferred tools in hunting impact and management studies in the Amazon (e.g., [20, 25, 26, 27]), for at least two important reasons: the low cost and equipment requirements for transect implementation, and the method’s suitability for the meaningful involvement of local and traditional communities in data collection [28].

Encounter-based methods often fail to detect animals confirmed to be present through sign data or casual observations in Neotropical forests [11, 22]. Failure to detect animals, if not accounted for through modeling and inference, may lead to underestimates of animal occurrence, abundance, density and range. Underestimation will be exacerbated in situations where hunting and other human activity causes behavioral shifts such as shyness and hiding in animals [29, 30, 31], decreasing detectability. Use of encounter data for density estimation and occupancy rests on the assumption that modeling is able to compensate for low detectability. However, density estimation cannot differentiate between actual rarity and apparent rarity due to wariness and hiding behavior, nor can it develop a non-zero estimate in situations where animals are simply not detected at all, unless it borrows detectability values (or probability of occurrence in the case of occupancy modeling) from another area.

From a conservation perspective, under-detection of animals and overestimation of rarity favor the precautionary principle, which has extended the “empty forest hypothesis” from areas with documented loss of animals to areas with predicted loss of animals [7, 9]. However, for communities that depend on wild meat for food and managers seeking to support sustainable, forest-based livelihoods, under-detection can result in unnecessarily restrictive management recommendations [32]. Here we use a large biogeographic field study from an Amazonian area about the size of Costa Rica to compare the accuracy of distance sampling and sign detection in describing the abundance of hunted and non-hunted vertebrates at very large landscape scales, using data sets collected by expert hunter-trackers. We provide detailed results for the lowland tapir (*Tapirus terrestris*) to illustrate the gaps in knowledge that can occur when visual encounter data alone are used.

## Methods

**Fig 1:**
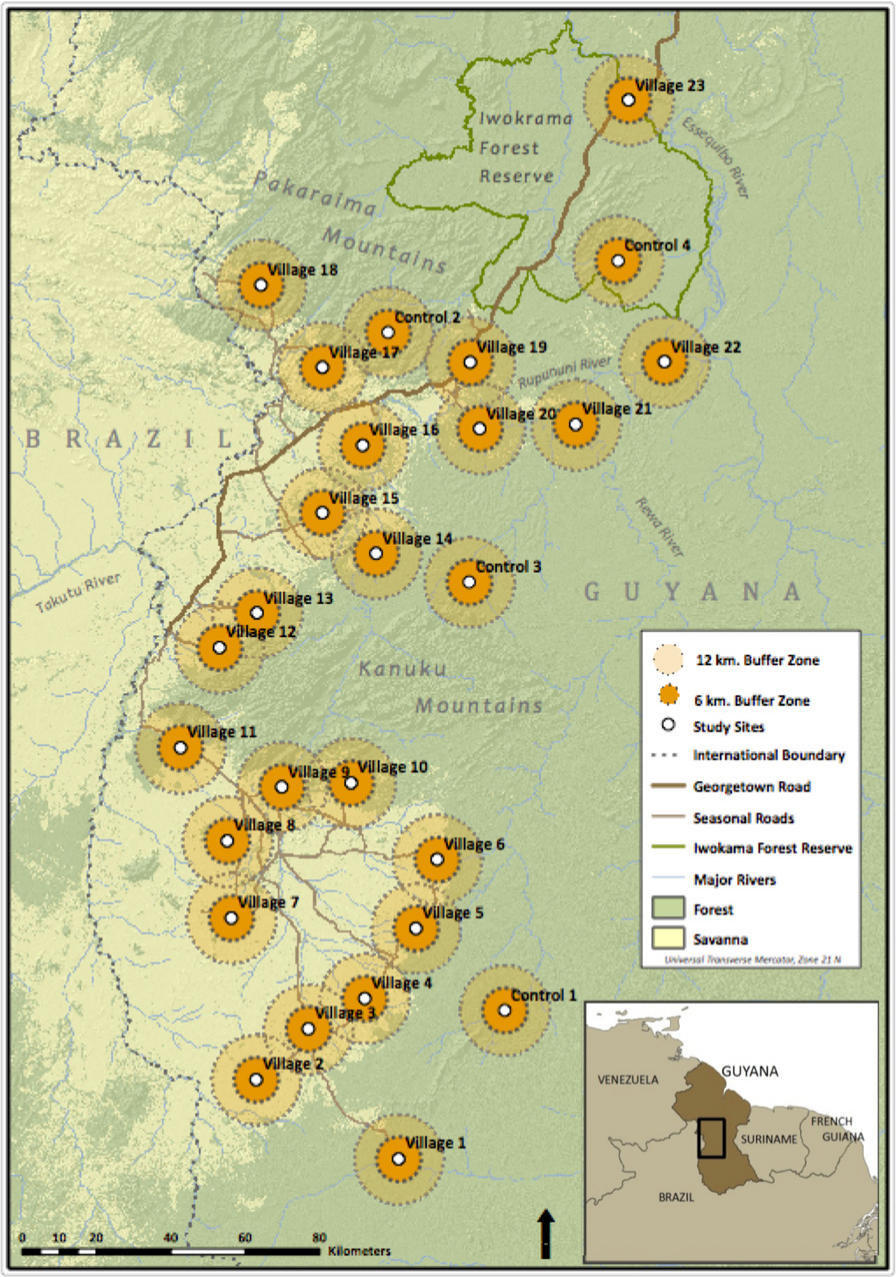
Map of the Guyana study region with village and control sites, and 0–12 km distance zones for transect placement (map adapted from [34]).

The study area in Guyana’s Rupununi region (Fig 1) encompasses forest and savannah ecosystems occurring across a range of elevations. It includes most of the traditional homelands and titled lands of approximately 20,000 Makushi and Wapishana people. All terrestrial ungulates, large rodents, large reptiles and some large birds are hunted in the region, although hunting levels vary among communities [33]. Primates are not eaten and are never or rarely collected [33, 34].

### Distance sampling and sign collection

Eight 4 km-long, straight-line transects were established around each of 23 village sites and 4 uninhabited control sites, for a total of 216 transects. Transects were placed in a stratified random fashion, divided equally between two distance zones (0–6 km and 6–12 km from the village (or from the site center in the case of uninhabited control areas)), in order to capture the full range of hunting intensities around communities. Transect placement was also stratified to avoid placement in areas where concentric 12 km sample areas of adjacent villages overlap. A 3 km minimum spacing between transects maximized independence for individuals or herds detected while still allowing for replication of transects within each distance zone (Fig 1). Transect length was selected to increase the probability that transects would traverse the home range of the farthest ranging species in the system (white-lipped peccaries, *Tayassu pecari* [35]). In rare situations, barriers were encountered that could not safely or effectively be surveyed (e.g., cliffs). In these instances, a transect turned to the right at a 90° angle, and continued until the 4 km end point.

From May 2007 to June 2010, both animal sign (predominantly tracks, but also feces, hair, carcasses or body parts, digging, burrows, eaten fruit or seeds, and browsed plants) and direct encounter data were collected monthly along transects. Encounter and sign data were collected on different days, separated by a period of one or two weeks, because each method requires different sampling techniques. For logistic reasons related to the large sample size and timing of transect implementation, the 27 study sites were incorporated in a time-staggered fashion into the project, such that by the end of the study individual sites had a minimum of 12 and a maximum of 38 months of data collection (median = 27 months). Cumulative distance walked on the 216 transects by the end of the study was greater than 40,000 km. Data were collected by indigenous hunters who were expert trackers and had been trained in transect sampling methodologies [34]. Training included the use of species guides and the application of sampling protocols; it was provided during one initial 6-day-long field course, followed by annual, 3-day-long retraining workshops designed to ensure consistency and accuracy in data collection. Standard distance sampling methods [36] were used for visual encounter data. Sign data were collected only within the one-meter center width of the entire transect. To avoid recounting the same sign during subsequent monthly sampling periods, only signs deemed by indigenous trackers to have been left within the three days preceding sampling were recorded. For a discussion on data quality control, see Luzar et al. [34]. Detailed vegetation classification along transects is described elsewhere [37]. Collection of sign and encounter data on the same transects during the same month ensured that identical vegetation was sampled by both techniques, eliminating this as a possible confounding variable.

### Kill data

Information on kills was collected from 23 indigenous communities from May 2007 to July 2010 and used to rank species by frequency of capture, a proxy for hunting pressure. Hunting surveys were administered weekly to every household by resident village technicians trained in interview techniques and data recording (see [34]). Information included species and number of individuals killed or collected in each hunting event. The location of each kill was plotted on a map. Kills in the area were made with bows and arrows (58%), shotguns (24%), machetes (8%), or with traps or other items (10%) [33]. The project was reviewed and approved by the University of Hawaii’s Committee on Human Studies. Informed consent for recording animal kills was provided verbally by participants. Consent was confirmed if the community member agreed to proceed. It was agreed with village and other indigenous leaders that data would be analyzed and reported anonymously unless village leaders provided exemption.

### Villages

Village population size varied from 122 to 1192 people. The predominant livelihood in all villages is subsistence hunting, fishing and farming [34]. No large-scale habitat degradation exists in the study area; a few villages practice small-scale selective timber extraction for local use [37].

### Species accumulation curves

A single detection event for sign or sighting determined species occurrence on a transect. To assess the risk of failing to detect species despite their occurrence, we constructed species accumulation curves for each method. Accumulation curves represent the number of transects on which the occurrence of a species is recorded, as a function of the number of times that the study area (all transects) is re-sampled. The number of re-sampling events (walks) required before a curve asymptotes indicates the level of effort necessary with each method to confidently assess species occurrence in the study area.

## Results

Accuracy in detection and accumulation curves for the two methods were compared using the set of mammals, reptiles and birds commonly hunted throughout the Amazon, even if they are not hunted in the Rupununi. We also compared method performance for 16 unhunted species (see below).

### Hunting Pressure

A total of 8,391 individuals from 127 species of mammals, birds, reptiles and amphibians were killed or collected at the 23 villages over 3 years (S1 Table). Thirty-one species with at least 20 individuals killed or harvested during the study provided an estimated harvest of 151,719 kg (S1 Table lists number of kills for all species and common and scientific names). The six native ungulate species contributed about a third of all individuals killed and 74% of the total biomass (Table 1). Lowland tapirs (*T*. *terrestris*), with 171 individuals killed, contributed more biomass than any other species (28%; 42,750 kg; Table 2, S1 Table). The two peccary species (*Tayassu pecari*, *Pecari tajacu)* contributed 26% of all biomass. The white-lipped peccary (*T*. *pecari*) was the third most commonly killed species (n = 908 individuals) and ranked second in terms of biomass (25,927 kilos), followed by the collared peccary (*P*. *tajacu;* n = 781; 13,681 kg). Three species of feral ungulates (pig, cow, water buffalo) provided an additional 3.46% percent of the biomass collected, an amount greater than that of all hunted birds and about equal to that of armadillos (*Dasypodidae*; Table 1). The number of individuals killed of three rodent species (agouti *Dasyprocta leporina*, paca *Cuniculus paca* and capybara *Hydrochoerus hydrochaeris*; ranked 1, 2 and 13^th^ in number killed, respectively) was equivalent to that of all six wild ungulate species; however, they contributed much less towards the total biomass (11.52% of the total biomass; Table 1). Birds contributed only 0.66% of biomass, less than any other hunted species groups. No or extremely few individuals were killed or collected of the 16 species classified as unhunted (Table 1, S1 Table).

**Table 1:** Biomass and number of individuals per species group or class killed or collected by hunters from May 2007 to July 2010.

Species Group (No. Species)	No. of Individuals Killed	Total Biomass (kg)	% of Individuals Killed	% of Kill Biomass
Native ungulates (6)	2812	112,807.97	33.51	74.35
Rodents (3)	2447	17,427.18	29.16	11.52
Reptiles (8)	972	3,879.89	11.59	5.26
Armadillos	952	5,364.94	11.35	3.54
Birds (9)	646	1,061.56	8.24	0.66
Unhunted Terrestrial Species (6)	77	471.00	0.86	0.31
Feral ungulates (3)	38	5,250.00	0.45	3.46
Primates (8)	34	168.22	0.42	0.09

**Table 2:** Kill rank for 32 native species of hunted and unhunted animals and best method for their detection. Also presented is the amount of effort needed for data to asymptote from sign transects and habitat for each species. + Indicates that sign is the better method for detection, = means the two methods are equally good and–indicates that encounter is the better method).

Taxa	Kill Rank by Biomass	Relative Detection Sign vs. Sight	Effort needed For Sign to Asymptote (N transects; N walks)	Habitat T = terrestrial, W = aquatic, A = arboreal
Lowland tapir	1	**+**	148 (6)	T
White-lipped peccary	2	**+**	90 (8)	T
White-tailed deer	3	**+**	100 (7)	T
Collared peccary	4	**+**	145 (8)	T
Red brocket deer	5	**+**	160 (7)	T
Paca	6	**+**	140 (15)	T
Capybara	7	**+**	20 (15)	T & W
Red-footed tortoise	8	**-**	27 (20)	T
Agouti	10	**=**	150 (10)	T
Long-nosed armadillo	11	**+**	155 (10)	T
Nine-banded armadillo	12	**+**	110 (20)	T
Yellow-footed tortoise	15	**-**	43 (25)	T
Amazonian brown brocket deer	17	**+**	130 (24)	T
Giant armadillo	21	**+**	120 (15)	T
Black Curassow	22	**=**	100 (7)	T & A
Naked-tailed armadillo	25	**+**	110 (18)	T
Giant anteater	26	**+**	160 (15)	T
Spider monkey	29	**-**	50 (19)	A
Coati	31	**=**	*	T & A
Accouchi	32	**-**	35 (8)	T
Howler monkey	34	**-**	70 (7)	A
Brown capuchin	35	**-**	20 (14)	A
Marail Guan	39	**-**	40 (30)	T & A
Tayra	40	**-**	70 (17)	T & A
Squirrel monkey	42	**-**	10 (18)	A
Wedge-capped capuchin	44	**-**	20 (7)	A
Golden-handed tamarin	46	**-**	9 (9)	A
Blue-throated piping guan	N	**-**	*	A
Bearded cuxiú	N	**-**	10 (17)	A
Guianan saki	N	**-**	5 (18)	A
Tamandua	N	**+**	68 (20)	T
Grison	N	**+**	15 (20)	T

N = none harvested

* did not asymptote

### Occurrence and distribution

#### Ungulates

**Fig 2:**
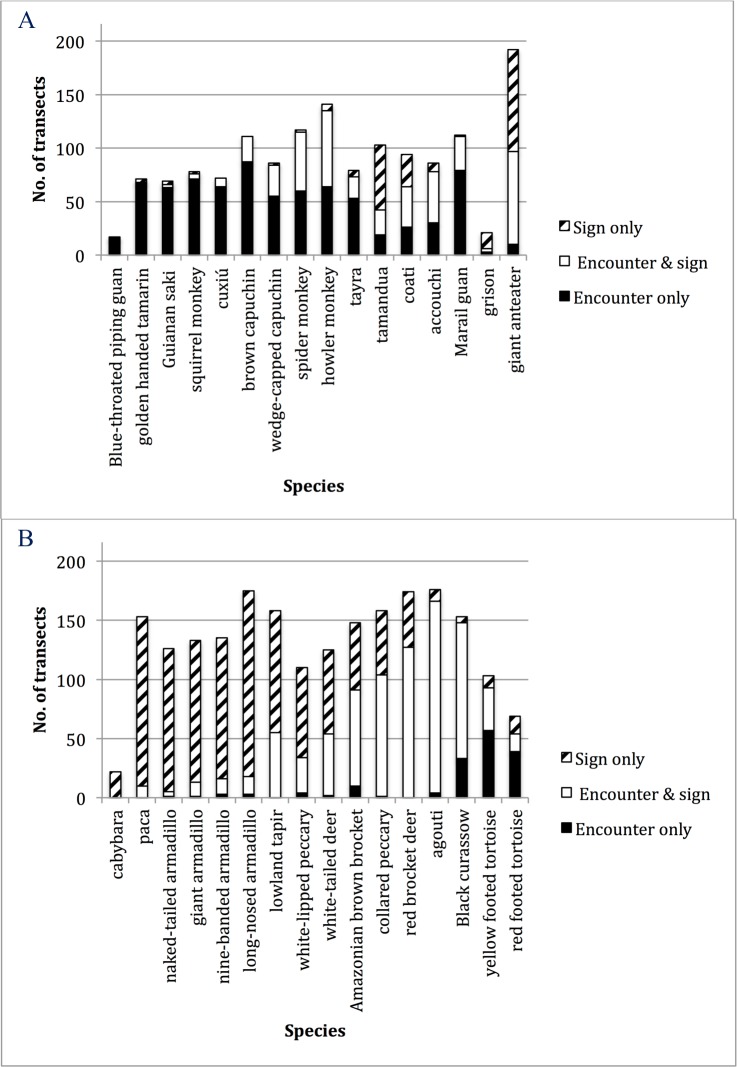
Number of transects, out of 216 total transects, on which sign, encounter, or both methods detected (A) hunted and (B) non-hunted species.

The encounter method failed to detect the six heavily hunted ungulates at 11–40% of the village and control sites where they were confirmed to occur on the basis of sign data (Table 3). At the transect level, the encounter method failed to detect these ungulates on 29–72% of transects where they were detected by sign (Fig 2A).

**Table 3:** Failure to encounter heavily hunted ungulate species at village and control site level.

Species	Sites with Encounters	Sites with Sign	Failure to Encounter
White-tailed deer	18	21	3
Amazonian brown brocket	22	25	3
Red brocket deer	23	27	4
White-lipped peccary	13	22	11
Collared peccary	23	25	2
Lowland tapir	16	25	9

#### Hunted, non-ungulate terrestrial species

Detection failure by encounter during diurnal transects was as expected high (~90%) for nocturnal species (e.g., *C*. *paca*, four armadillo species) and for the diurnal rodent the capybara (*H*. *hydrochaeris*; 100%), but low for agouti (*D*. *leporina*), tortoises (*Geochelone* spp.) and the one game bird (Black Curassow *Crax alector*; Fig 2A and 2B).

#### Non-hunted, terrestrial species

Of the terrestrial non-hunted species, the giant anteater (*Myrmecophaga tridactyla*), coati (*Nasua nasua*), tamandua (*Tamandua tetradactyla*) and grison (*Galictis vittata)* had higher detection rates with sign (Fig 2B; Table 2). In contrast, the tayra (*Eira barbara*) was more frequently detected visually.

#### Non-hunted, arboreal species

The Blue-throated Piping Guan (*Pipile cumanensis*) was encountered on 14 transects, but detected on only one transect by sign. The rarely hunted Marail Guan (*Penelope marail*) was also better detected by encounters. Black spider monkeys (*Ateles paniscus*) were encountered on 110 transects, but their sign was observed on only 60 transects; similarly, the bearded cuxiú (*Chiropotes chiropotes*) was encountered on 70 transects but their sign on only 8 transects. This pattern repeated for the other 6 primate species found in the region (Fig 2B)

Sign data thus performed better or equally well as encounter data for detecting hunted terrestrial rodents and birds where they occur, and outperformed encounter data for hunted ungulates, nocturnal species, and four non hunted terrestrial species (Fig 2A and 2B; Table 2). As expected sign data underperformed encounter data for arboreal species. Nevertheless, on transects where a terrestrial species was detected by both methods, the frequencies of encounter derived from each method were positively correlated (Pearson’s correlation coefficient, r = .138 to .627, p = .0000 to .0427; Table 4).

**Table 4:** Correlations between number of wildlife sightings and number of sign on transects where both occur, for the focal ungulate species and a few representative species from the other guilds. Total transects available for use where 216, with data collected from April 2007 to June 2010.

Species	Pr, r value	Pr, P value
White-tailed deer	0.425	0.0000
Amazonian brown brocket	0.506	0.0000
Red brocket deer	0.542	0.0000
White-lipped peccary	0.260	0.0001
Collared peccary	0.625	0.0000
Lowland tapir	0.455	0.0000
Accouchi	0.334	0.0000
Agouti	0.627	0.0000
Paca	0.627	0.0000
Black Curassow	0.587	0.0000
Marail Guan	0.193	0.0045
Long nosed armadillo	0.138	0.0427

Pr = Pearson’s test. Note the lower r-values for nocturnal and arboreal species.

### Accumulation curves

**Fig 3:**
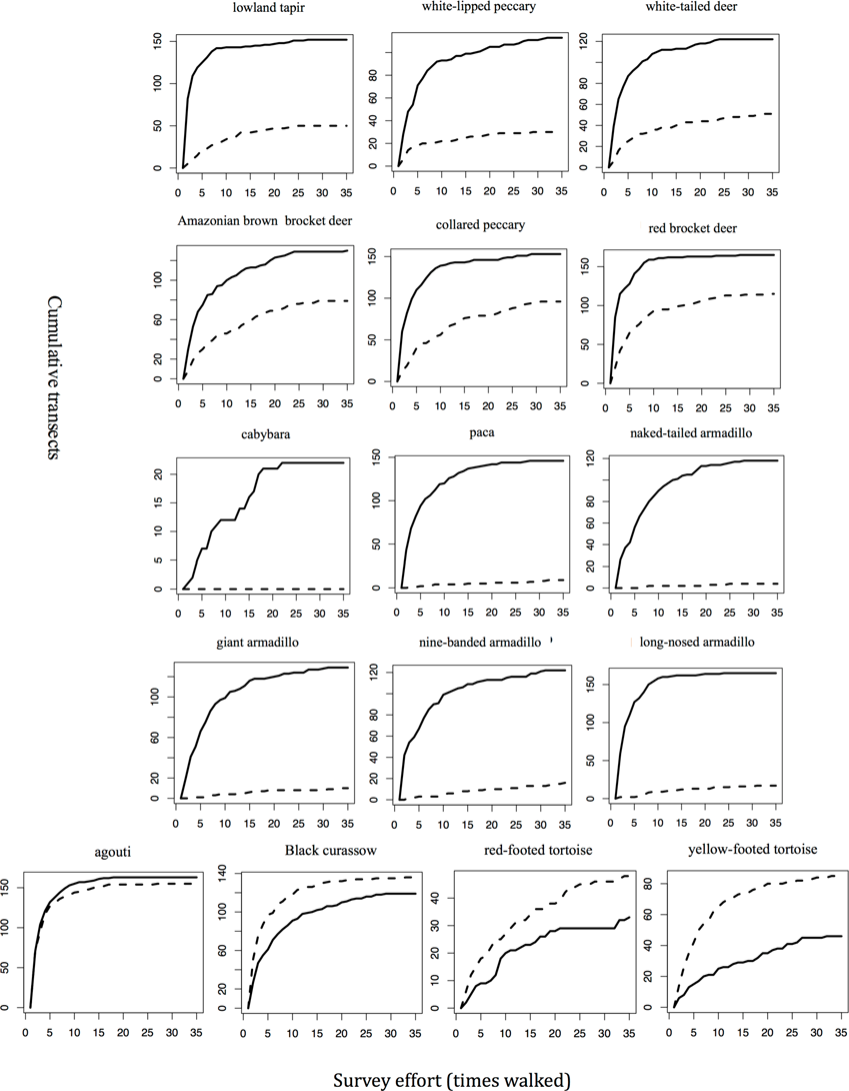
Accumulation rates for sign vs. encounters (% of transects on which they are detected as the study progresses) for 16 hunted species. Effort is reported in terms of cumulative survey months and equivalent kilometers walked. The Y axis represents the number of transects with presence recorded. The X axis represents the number of times transects were walked (i.e., number of times you have to resample the study area). A solid line represents sign and the hatched line encounters.

**Fig 4:**
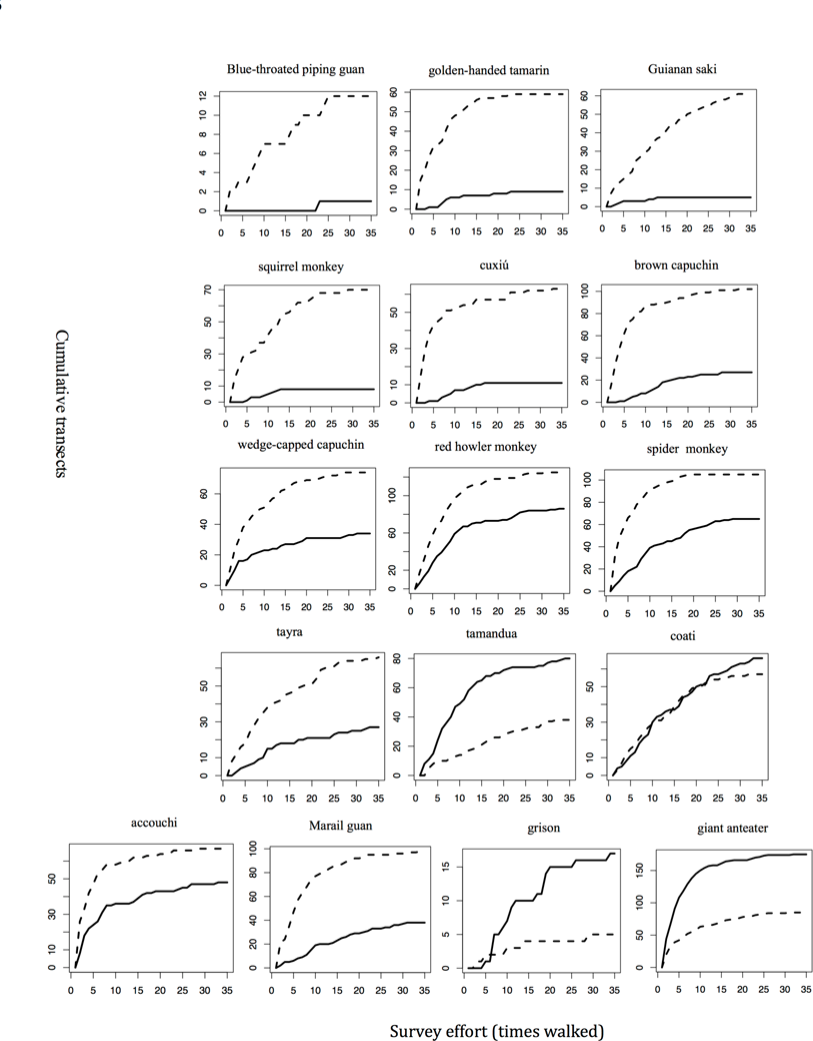
Accumulation rates for sign vs. encounters (% of transects on which they are detected as the study progresses) for 16 unhunted species. Effort is reported in terms of cumulative survey months and equivalent kilometers walked. The Y axis represents the number of transects with presence recorded. The X axis represents the number of times transects were walked (i.e., number of times you have to resample the study area). A solid line represents sign and the hatched line encounters.

For all 32 hunted and non hunted species analyzed, the number of transects walked during the study was sufficient for the data to asymptote with at least one of the methods (Figs 3 and 4). Two types of information were extracted from the accumulation curves: (1) the number of transects on which the species was detected when the curve reached an asymptote with each of the methods; and (2) the effort (number of re-sampling events) needed for the accumulation curve to reach an asymptote, indicating that the species had been detected in all places where it occurred during the study.

For 14 of 16 native hunted species, accumulation curves for sign reached an asymptote on more transects with fewer or a similar number of walks as for encounters (Table 2), indicating not only that the method is more likely to detect the species, but that it does so with a similar or lower level of effort as encounters. On the other hand, encounters reached an asymptote on more transects and with fewer walks for the two hunted tortoise species (ranked 8 and 15 in kill biomass). Encounters and sign performed similarly well for the hunted agouti (*D*. *leporina*) and the mostly terrestrial Black Curassow (*C*. *alector*; ranked 10 and 22 in kill biomass, respectively).

Armadillos, paca (*C*. *paca*) and the six native ungulates, all heavily hunted species, achieved an asymptote of detection on 90 to 150 transects at 8 to 25 walks with sign data (Table 2, Fig 3). However, three of the ungulates—red brocket deer (*Mazama americana*), white-lipped peccary (*T*. *pecari*), and lowland tapir (*T*. *terrestris*)—reached an asymptote on fewer transects and less walks with encounter data than with sign data (i.e., they had not been detected on all transects where they occurred when the method indicated complete detection). Paca (*C*. *paca*), capybara *(H*. *hydrochaeris)* and armadillos reached asymptote with sign but not encounters. For the abundant and commonly hunted agouti (*D*. *leporina*), sign and encounter data were equally robust, whereas for the non-hunted and also large accouchi rodent (*Myoprocta acouchy*), encounter data were more robust. Encounter and sign data were almost equally robust for the heavily hunted Black Curassow (*C*. *alector*), whereas the non-hunted Blue-throated Piping Guan (*P*. *cumanensis*) and the eight non-hunted primate species attained an asymptote with lower effort and at fewer transects with encounter data (Fig 4).

### Tapir kill rates

**Fig 5:**
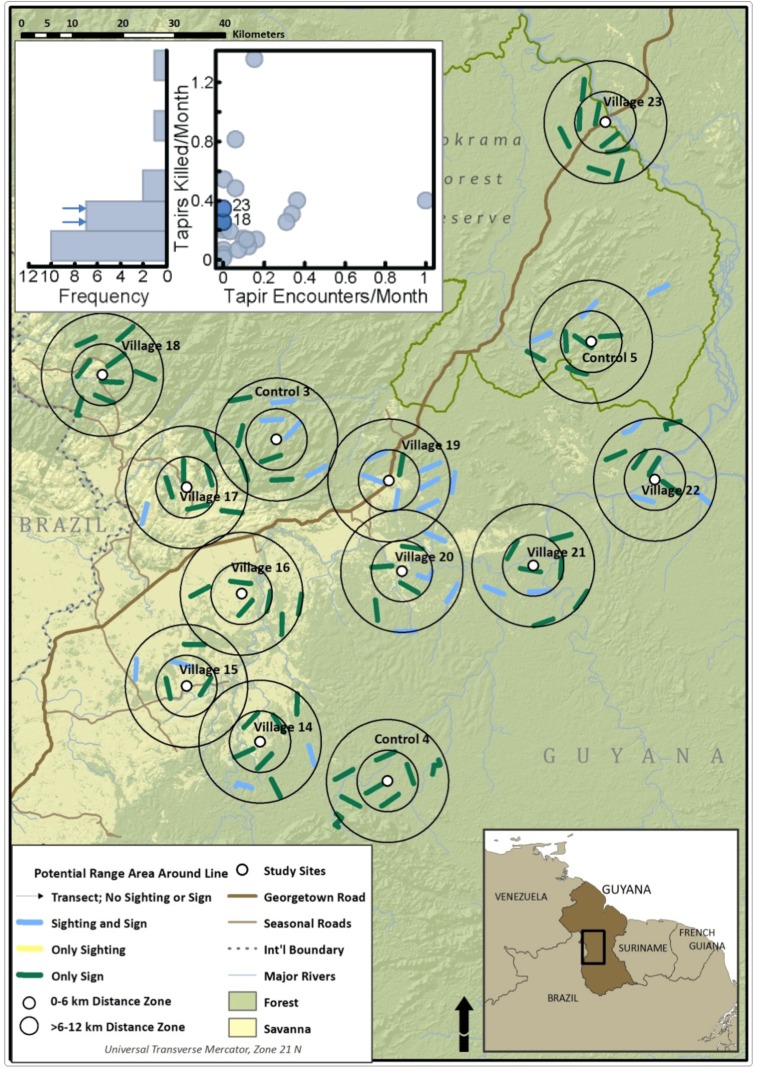
Tapir occupancy of transects around northern village study sites (map adapted from [34]). Inset graphs show tapir encounters and kills for 13 sites, with village numbers 18 and 23 highlighted.

Lowland tapirs (*T*. *terrestris*) were detected by sign at 21 of 23 villages and all control sites, but were never visually encountered at 10 of these villages and 1 control site. They were detected by sign on 151 transects, but were never encountered on 99 of transects where they were detected by sign. And yet, at villages where they were never encountered tapirs were killed at rates similar to those at other villages in the region where they were encountered [33]: mapping of the transect data for the northern region of the study area (Fig 5, for tapirs) illustrates the mesoscale gaps in distribution that result from encounter data and can affect our understanding of population structure. Tapir were killed at villages 18 and 23, where they were never encountered but were frequently detected by sign (Fig 5), and at kill rates (0.26 and 0.34 tapirs killed per month, respectively; Fig 3 inset) that overlapped those of other villages in the region where tapirs were directly encountered (range 0.14 to 0.48).

## Discussion

Given the mandate in most Amazonian countries for co-management of resources on multiple use protected areas and indigenous lands [9, 38], the recognized need to secure the role of wild meat for the food security of poor and isolated human populations in the tropics [39], and the continued interest of researchers in understanding the consequences of defaunation for forest regeneration [40, 41], it is of critical importance that robust, accurate methodologies be used to assess hunted vertebrate populations and inform their management. Conclusions about animal abundance and distribution patterns based on potentially flawed data sets will compromise our understanding of animal population dynamics in the Neotropics and, by extension, the competence of management decision-making.

Despite the increasing popularity of camera traps, and the integration of multiple data sources in occupancy modeling [42], distance sampling remains the most commonly used method for assessing wildlife populations for conservation and management purposes in the Amazon. The use of linear transects for wildlife sampling will, justifiably, remain in wide use for the foreseeable future, especially as more remote lands inaccessible to research scientists are brought under monitoring and management regimes by their local inhabitants [43]. Although occupancy methods will be important in better describing the distribution of species, it is unlikely that they will provide sufficient information [44] for rapid-response adaptive management—the adaptation of management rules using continuous monitoring data. Occupancy of a given site by 1 versus 50 tapirs at any one time is irrelevant for occupancy modeling, but highly significant for setting harvest rates and hunting zones for a local community. Our results show that, even with massive sampling effort, it is not possible to increase encounter-based detectability for many commonly hunted, behaviorally non-cryptic (when unhunted) Neotropical mammals (tapir, both species of peccary, three species of deer) to levels that reflect their true abundance or even occurrence; for these species, sign-based methods more reliably detected the presence of animals and hold promise as reliable indices—or even direct measures—of abundance. In other cases, such as for the agouti, both methods similarly describe the distribution of animals on the landscape, with each method missing the species on only a few transects where it was detected by the other method (Fig 2A). Inspecting the accumulation curves also shows that both methods are equally efficient at detecting the species and describing its distribution or occupancy.

The decision of which method(s) to use for animal surveys or monitoring of hunting impacts will depend on the focal species’ habits (arboreal vs. terrestrial, diurnal vs. nocturnal, behaviorally responsive to hunting or not). Shifts in behavior in response to hunting are extensively documented for many game species (e.g., [45, 46, 47, 48]). For nocturnal and hunted animals, such as armadillos and paca, the lack of visual encounters during daytime transects cannot be ascribed to shifts in behavior in response to hunting pressure without further experimental study. The absence of difference between encounter and sign data for agoutis suggests the absence of hiding behavior, perhaps due to the animals’ very small home range. Hiding behavior, however, could be one important explanation for the low visual detection of the heavily hunted, behaviorally non-cryptic ungulates. Other factors affecting detectability will include abundance levels, cryptic behavior, and observer–environment interaction, among others. It will be challenging but important to assess the relationship between abundance of tracks and abundance of animals for these species, as hiding behavior may affect frequency of sign as well as frequency of visual encounters.

The management implications of method selection are illustrated by the case of the tapir in this study. A conclusion of local tapir extinction would have been reached at the two villages with no tapir sightings, if abundances were estimated on the basis of encounters only and sign had not been recorded. Given the location of one of these villages within a multiple use protected area, results pointing to the absence of tapirs would likely result in restrictions on hunting-based livelihoods. Similarly, on the basis of encounter data alone, the impacts of sustainable logging on tapirs in the multiple use zones of this reserve would have been overestimated, resulting in unnecessary restrictions on sustainable forestry activities. Inferences about the level of seed dispersal services provided by tapirs in different locations of this protected area would also be incorrect if based only on encounter data.

An important consideration in method selection is also the efficiency (more rapid data accumulation) of sign sampling over visual encounters, which would enable abundance estimates for species that occur at low densities (e.g., tapir) or exhibit cryptic behavior (e.g., the unhunted giant anteater *M*. *tridactyla*, tamandua *T*. *tetradactyla* and grison *G*. *vittata* in this study). Encounter rates for lowland tapirs wherever they are studied are too low to estimate local densities [17] without extensive replication efforts (i.e., repeated transect sampling). Even for our three-year study, encounter data from all transects and sites would have to be pooled to estimate densities using distance sampling methodologies; such pooling is inappropriate given that tapir densities are expected to differ across the diversity of habitats and hunting pressures in this landscape. The resulting estimated density would not be accurate for most locations in the study area, and a number for the entire study area would be meaningless from a management perspective. Landscape scale management decisions and coordination for management among different villages would not be possible on the basis of encounter data alone, but could be achieved on the basis of sign data; such data can be continually obtained and yield reliable information on changes in both abundance and occupancy over time in response to management interventions [43].

Although many studies do use sign in an ad hoc manner to assess the abundance of animals that are difficult to detect with encounters (e.g. nocturnal animals such as armadillos; [49], there has been little effort in the Neotropics to develop field protocols and statistical methods that enable abundance or density estimation from sign data [50]. The strong correlation between sign and sighting data in this study, as well as the steepness of the accumulation curves, indicates that for many Neotropical species it may be feasible to use sign data as a meaningful index of animal density and/or abundance. Efforts invested in calibration between sign data and true abundance, combined with long-term data collection, have enabled extensive use of sign data as relative or absolute measures for vertebrate abundance in North America, Europe, and Africa [51, 52, 53], where such data have become critical to ecological studies and decision-making regarding hunting quotas [53]. Such calibration has rarely been attempted in Neotropical forests (but see [54]). Such calibration will be especially important in allowing ecologically accurate interpretation of occupancy modeling results, given the increasing interest in using multiple sources of detection data to describe animal abundances and the low cost and high efficiency of obtaining sign data (more rapidly obtained).

The important contribution of locally collected and interpreted data for local community-based governance and rapid management decision-making is an additional and important benefit of sign data [28, 55]. The efficiency of data collection by sign—sufficient data for statistically reliable analyses collected in a shorter period of time than encounter data—points to local trackers as effective environmental monitors, who can monitor local trends in animal populations and provide up-to-date input to local co-management decisions.

Our results demonstrate that direct encounter data chronically under-detect the presence of most, but not all, hunted species in Amazonian ecosystems, yielding inaccurate assessments of animal abundance, density, spatial distribution, and the impact of hunting. Policy responses to these assessments can include restrictions on hunting within multiple use protected areas and indigenous lands, inclusion of incentives to reduce dependence on wild meat in environmentally conditioned poverty alleviation payments, and rejection of the contribution of indigenous and local communities to biodiversity conservation. Such policies can negatively impact food security for subsistence peoples, while contributing little to conservation goals.

## Supporting Information

S1 Table The species, their biomass and number collected from May 2007 to June 2010. (DOCX)
