# Supplementary material for: Line Transect Surveys Underdetect Terrestrial Mammals: Implications for the Sustainability of Subsistence Hunting
Source: PLoS One. 2016 Apr 13;11(4):e0152659. doi: 10.1371/journal.pone.0152659 (PMC4830449; doi:10.1371/journal.pone.0152659)
Supplement: S1 Table — (DOCX) [file pone.0152659.s001.docx]

S1 Table: The species, their biomass and number collected from May 2007 to June 2010 in 23 villages of the Rupununi, Guyana.

|  | Rank | Rank | % | % | Total | Total | Estimated Ind. | Ind. Weight |
| --- | --- | --- | --- | --- | --- | --- | --- | --- |
| Taxa | Biomass | No. Ind. | Biomass | Ind. | Biomass | No. Ind. | weight (kg) | Source Reference |
| Lowland tapir (*Tapirus terrestris*) | 1 | 14 | 28.18 | 2.04 | 42,750 | 171 | 250 | 56 |
| White-lipped peccary (*Tayassu pecari*) | 2 | 3 | 17.09 | 10.82 | 25,927 | 908 | 28.5 | 57 |
| White-tailed deer (*Odocoileus virginianus*) | 3 | 7 | 11.71 | 5.29 | 17,760 | 444 | 40 | 56 |
| Collared peccary (*Pecari tajacu*) | 4 | 4 | 9.02 | 9.31 | 13,681 | 781 | 17.52 | 58 |
| Red brocket deer (*Mazama americana*) | 5 | 8 | 7.53 | 5.22 | 11,426 | 438 | 26.1 | 56 |
| Paca (*Cuniculus paca*) | 6 | 2 | 6.14 | 13.48 | 9,308 | 1131 | 8.23 | 56 |
| Capybara (*Hydrochoerus hydrochaeris*) | 7 | 13 | 3.76 | 2.16 | 5,702 | 181 | 31.5 | 56 |
| Red footed tortoise (*Geochelone carbonaria*) | 8 | 6 | 1.85 | 5.59 | 2,809 | 469 | 5.99 | 59 |
| Feral pig (*Sus scrofa*) | 9 | 23 | 1.68 | 0.41 | 2,550 | 34 | 75 | 59 |
| Agouti (*Dasyprocta leporina*) | 10 | 1 | 1.59 | 13.53 | 2,418 | 1135 | 2.13 | 60 |
| Long nosed armadillo (*Dasypus kappleri*) | 11 | 10 | 1.55 | 2.94 | 2,346 | 247 | 9.5 | 59 |
| Nine-banded armadillo (*Dasypus novemcinctus*) | 12 | 5 | 1.25 | 6.38 | 1,896 | 536 | 3.54 | 59 |
| Feral cow (*Bos taurus*) | 13 | 44 | 1.19 | 0.04 | 1,800 | 3 | 600 | 59 |
| Jaguar (*Panthera onca*) | 14 | 29 | 1.09 | 0.26 | 1,650 | 22 | 75 | 59 |
| Yellow footed tortoise (*Geochelone denticulate*) | 15 | 9 | 1.06 | 3.26 | 1,608 | 273 | 5.88 | 59 |
| Spectacled caiman (*Caiman crocodilus*) | 16 | 24 | 0.87 | 0.39 | 1,320 | 33 | 40 | 59 |
| Amazonian brown brocket deer (*Mazama nemorivaga*) | 17 | 17 | 0.80 | 0.84 | 1,050 | 70 | 18 | 61 |
| Giant river turtle (*Podocnemis expansa*) | 18 | 31 | 0.61 | 0.24 | 920 | 20 | 46 | 59 |
| Feral water buffalo (*Bubalus bubalis*) | 19 | 46 | 0.59 | 0.01 | 900 | 1 | 900 | 59 |
| Dwarf caiman *Paleosuchus* spp*.*) | 20 | 16 | 0.49 | 1.28 | 749 | 107 | 7 | 59 |
| Giant armadillo (*Priodontes maximus*) | 21 | 30 | 0.45 | 0.25 | 677 | 21 | 32.5 | 59 |
| Black Curassow (*Crax alector*) | 22 | 11 | 0.44 | 2.57 | 670 | 216 | 3.1 | 59 |
| Anaconda *Eunectes* sp. | 23 | 36 | 0.30 | 0.12 | 450 | 10 | 45 | 59 |
| Cougar (*Puma concolor*) | 24 | 37 | 0.30 | 0.12 | 450 | 10 | 45 | 59 |
| Naked-tailed armadillo (*Cabassous unicinctus*) | 25 | 15 | 0.29 | 1.77 | 446 | 149 | 3 | 59 |
| Giant anteater (*Myrmecophaga tridactyla*) | 26 | 38 | 0.20 | 0.11 | 306 | 9 | 34 | 59 |
| Muscovy duck (*Cairina moschata*) | 27 | 21 | 0.15 | 0.52 | 220 | 44 | 5 | 59 |
| Green iguana (*Iguana iguana*) | 28 | 25 | 0.07 | 0.39 | 99 | 33 | 3 | 59 |
| Black spider monkey (*Ateles paniscus*) | 29 | 35 | 0.07 | 0.13 | 99 | 11 | 9 | 59 |
| Green-winged macaw (*Ara chloroptera*) | 30 | 18 | 0.06 | 0.74 | 84 | 62 | 1.35 | 59 |
| Coati (*Nasua nasua*) | 31 | 32 | 0.04 | 0.17 | 56 | 14 | 4 | 59 |
| Accouchi (*Myoprocta acouchy*) | 32 | 22 | 0.03 | 0.48 | 52 | 40 | 1.3 | 56 |
| White faced whistling duck (*Dendrocygna viduata*) | 33 | 19 | 0.03 | 0.57 | 41 | 48 | 0.85 | 59 |
| Red howler monkey (*Alouatta seniculus*) | 34 | 42 | 0.02 | 0.06 | 30 | 5 | 6 | 59 |
| Brown capuchin (*Cebus apella*) | 35 | 39 | 0.02 | 0.10 | 28 | 8 | 3.5 | 59 |
| Small river turtle (unknown species) | 36 | 26 | 0.02 | 0.32 | 27 | 27 | 1 | estimate JMVF |
| Southern tamandua (*Tamandua tetradactyla)* | 37 | 40 | 0.02 | 0.07 | 27 | 6 | 4.5 | 59 |
| Scarlet macaw (*Ara macao*) | 38 | 33 | 0.01 | 0.17 | 18 | 14 | 1.3 | 62 |
| Marail guan (*Penelope marail*) | 39 | 34 | 0.01 | 0.17 | 15 | 14 | 1.1 | 62 |
| Tayra (*Eira barbara*) | 40 | 45 | 0.01 | 0.04 | 15 | 3 | 5 | 59 |
| Red-bellied macaw (*Orthopsittaca manilatus*) | 41 | 28 | 0.00 | 0.46 | 11.7 | 39 | 0.3 | 59 |
| Squirrel monkey (*Saimiri sciureus*) | 42 | 41 | 0.00 | 0.07 | 6 | 6 | 1.05 | 56 |
| Chestnut-bellied seed finch (*Sporophila angolensis*) | 43 | 12 | 0.00 | 2.42 | 4 | 203 | 0.02 | JMVF estimate |
| Wedge-capped capuchin (*Cebus olivaceus*) | 44 | 47 | 0.00 | 0.01 | 3 | 1 | 3 | 59 |
| Brown-throated parakeet (*Aratinga pertinax*) | 45 | 27 | 0.00 | 0.27 | 2 | 23 | 0.1 | 62 |
| Golden handed tamarin (*Saguinus midas*) | 46 | 43 | 0.00 | 0.05 | 2 | 4 | 0.48 | 59 |
| Seed eater (unknown species) | 47 | 20 | 0.00 | 0.54 | 1 | 45 | 0.02 | 62 |
| Red-shouldered macaw (*Diopsittaca nobilis*) |  |  | nc | nc |  | 19 |  |  |
| Stingray (unknown species) |  |  | nc | nc |  | 16 |  |  |
| Spix's guan (*Penelope jacquacu*) |  |  | nc | nc |  | 15 |  |  |
| Little chachalaca (*Ortalis motmot*) |  |  | nc | nc |  | 14 |  |  |
| Macaw (unknown species) |  |  | nc | nc |  | 13 |  |  |
| White-tipped dove (*Leptotila verreauxi*) |  |  | nc | nc |  | 14 |  |  |
| Yellow-crowned parrot (*Amazona ochrocephala*) |  |  | nc | nc |  | 16 |  |  |
| Painted parakeet (*Pyrrhura picta*) |  |  | nc | nc |  | 11 |  |  |
| Crested bobwhite (*Colinus cristatus*) |  |  | nc | nc |  | 13 |  |  |
| Great tinamou (*Tinamus major*) |  |  | nc | nc |  | 10 |  |  |
| Plain-breasted ground dove (*Columbina minuta*) |  |  | nc | nc |  | 8 |  |  |
| Tegu lizard (*Tupinambis teguixin*) |  |  | nc | nc |  | 8 |  |  |
| Blue-grey tanager (*Thraupis episcopus*) |  |  | nc | nc |  | 7 |  |  |
| Pigeon (unknown species) |  |  | nc | nc |  | 7 |  |  |
| Crab-eating fox (*Cerdocyon thous*) |  |  | nc | nc |  | 6 |  |  |
| Grey-winged trumpeter (*Psophia crepitans*) |  |  | nc | nc |  | 6 |  |  |
| Ocelot (*Leopardus pardalis*) |  |  | nc | nc |  | 6 |  |  |
| Rattlesnake (*Crotalus durissus*) |  |  | nc | nc |  | 6 |  |  |
| Black-bellied whistling duck (*Dendrocygna autumnalis*) |  |  | nc | nc |  | 5 |  |  |
| Dove (unknown species) |  |  | nc | nc |  | 5 |  |  |
| River turtle (unknown species) |  |  | nc | nc |  | 5 |  |  |
| Blue and yellow macaw (*Ara ararauna*) |  |  | nc | nc |  | 4 |  |  |
| crab-eating raccoon (*Procyon cancrivorus*) |  |  | nc | nc |  | 4 |  |  |
| Duck (unknown species) |  |  | nc | nc |  | 4 |  |  |
| Hawk (unknown species) |  |  | nc | nc |  | 4 |  |  |
| Unknown species |  |  | nc | nc |  | 4 |  |  |
| Pale-vented pigeon (*Patagioenas cayennensis*) |  |  | nc | nc |  | 4 |  |  |
| Ruddy pigeon (*Patagioenas subvinacea*) |  |  | nc | nc |  | 4 |  |  |
| Savanna hawk (*Buteogallus meridionalis*) |  |  | nc | nc |  | 4 |  |  |
| Black-tailed hairy dwarf porcupine (*Coendou melanura*) |  |  | nc | nc |  | 3 |  |  |
| Blue-throated piping guan (*Pipile cumanensis*) |  |  | nc | nc |  | 3 |  |  |
| Eared dove (*Zenaida auriculata*) |  |  | nc | nc |  | 3 |  |  |
| Margay cat (*Leopardus wiedii*) |  |  | nc | nc |  | 3 |  |  |
| Neotropical cormorant (*Phalacrocorax brasilianus*) |  |  | nc | nc |  | 3 |  |  |
| Orange winged amazon parrot (*Amazona amazonica*) |  |  | nc | nc |  | 4 |  |  |
| Owl (unknown species) |  |  | nc | nc |  | 3 |  |  |
| Parrot (unknown species) |  |  | nc | nc |  | 3 |  |  |
| Toucan (unknown species) |  |  | nc | nc |  | 4 |  |  |
| Wood turtle (unknown species) |  |  | nc | nc |  | 3 |  |  |
| Birds (unkown species) |  |  | nc | nc |  | 2 |  |  |
| Buff-necked ibis (*Theristicus caudatus*) |  |  | nc | nc |  | 3 |  |  |
| Bushmaster (*Lachesis muta*) |  |  | nc | nc |  | 2 |  |  |
| Chicken hawk (unknown species) |  |  | nc | nc |  | 2 |  |  |
| Crestless curassow (*Mitu tomentosum*) |  |  | nc | nc |  | 2 |  |  |
| Deer (unknown species) |  |  | nc | nc |  | 2 |  |  |
| Jabiru (*Jabiru mycteria*) |  |  | nc | nc |  | 2 |  |  |
| Limpkin (*Aramus guarauna*) |  |  | nc | nc |  | 2 |  |  |
| Pale-throated three-toed sloth (*Bradypus tridactylus*) |  |  | nc | nc |  | 2 |  |  |
| Red-throated caracara (*Ibycter americanus*) |  |  | nc | nc |  | 2 |  |  |
| Snake (unknown species) |  |  | nc | nc |  | 2 |  |  |
| Tinamou (unknown species?) |  |  | nc | nc |  | 2 |  |  |
| Water turtle (unknown species) |  |  | nc | nc |  | 2 |  |  |
| Violaceous jay (*Cyanocorax violaceus)* |  |  | nc | nc |  | 1 |  |  |
| Red-tailed boa (*Boa constrictor)* |  |  | nc | nc |  | 1 |  |  |
| Brazilian teal (*Amazonetta brasiliensis*) |  |  | nc | nc |  | 1 |  |  |
| Bearded cuxiú |  |  | nc | nc |  | 1 |  |  |
| Bush deer (small) (unkown species) |  |  | nc | nc |  | 1 |  |  |
| Channel-billed toucan (*Ramphastos vitellinus*) |  |  | nc | nc |  | 1 |  |  |
| Common opossum (*Didelphis marsupialis*) |  |  | nc | nc |  | 1 |  |  |
| Crested caracara (*Caracara cheriway*) |  |  | nc | nc |  | 1 |  |  |
| Crested oropendola (*Psarocolius decumanus*) |  |  | nc | nc |  | 1 |  |  |
| Eagle (unknown species?) |  |  | nc | nc |  | 1 |  |  |
| Guyana side-necked turtle (*Phrynops tuberosus*) |  |  | nc | nc |  | 1 |  |  |
| Maguari stork (*Ciconia maguari*) |  |  | nc | nc |  | 1 |  |  |
| Mealy parrot (*Amazona farinosa*) |  |  | nc | nc |  | 1 |  |  |
| Neotropical otter (*Lontra longicaudis*) |  |  | nc | nc |  | 1 |  |  |
| Red-billed toucan (*Ramphastos tucanus*) |  |  | nc | nc |  | 1 |  |  |
| Scorpion mud turtle (*Kinosternon scorpioides*) |  |  | nc | nc |  | 1 |  |  |
| Sparrow (*Passer domesticus*) |  |  | nc | nc |  | 1 |  |  |
| Tawatawa (unknown species) |  |  | nc | nc |  | 1 |  |  |
| Undulated tinamou (*Crypturellus undulatus*) |  |  | nc | nc |  | 1 |  |  |
| White necked heron (*Ardea cocoi*) |  |  | nc | nc |  | 1 |  |  |
| White-faced spiny tree rat (*Echimys chrysurus*) |  |  | nc | nc |  | 1 |  |  |
| Totals |  |  |  |  | 151,719 | 8390 |  |  |

nc=unhunted (<20 killed), not calculated

**References**

*56* Eisenberg JF. Mammals of the Neotropics. v. 1. The northern neotropics: Panama, Colombia, Venezuela, Guyana, Suriname, French Guiana. Chicago: Chicago University Press; 1989.

57 Fragoso JMV. Home range and movement Patterns of White-lipped Peccary (*Tayassu pecari*) herds in the Northern Brazilian Amazon. Biotropica. 1998;30:458-69.

*58* Fragoso JMV. Large mammals and the community dynamics of an Amazonian rain forest. Ph.D. Dissertation, The University of Florida. 1994.

*59* Wikipedia. 2016. (cited 10 January 2016). Available: <https://en.wikipedia.org/wiki/Main_Page>.

*60* Silvius KM, Fragoso J. Red-rumped agouti (*Dasyprocta leporina)* home range use in an Amazonian forest: implications for the aggregated distribution of forest trees. Biotropica. 2003;35:74-83.

*61* Rossi RV, Bodmer RE, Barbanti Duarte JM, Trovati RG. Amazonian brown brocket deer *Mazama nemorivaga (Cuvier 1817)*. In: Duarte JMB, Gonzalez S, editors. Neotropical cervidology: biology and medicine of Latin American deer. Brazil: Funep; 2010. p. 202-210.

*62* Oiseaux net. 2016. (cited 10 December 2015). Available: http://www.oiseaux.net/
